# Highly Sensitive Parylene C‐Based Flexible Pressure Sensors for Wearable Systems

**DOI:** 10.1002/smsc.202500081

**Published:** 2025-05-14

**Authors:** Zhao Wang, Bhavani P. Yalagala, Hadi Heidari, Andrew Feeney

**Affiliations:** ^1^ Centre for Medical and Industrial Ultrasonics (C‐MIU) James Watt School of Engineering University of Glasgow Glasgow G12 8QQ UK; ^2^ Microelectronics Lab (meLAB) Group James Watt School of Engineering University of Glasgow Glasgow G12 8QQ UK; ^3^ Present address: James Watt School of Engineering University of Glasgow, University Avenue Glasgow G12 8QQ UK

**Keywords:** biocompatible, flexible pressure sensors, Parylene C, piezoelectrics, real time, smart gloves, wireless

## Abstract

Flexible pressure sensors based on piezoelectric materials are being extensively investigated, but the potential of Parylene C in this application has rarely been explored, even though it has superior electrical insulation, excellent chemical inertness, flexibility, biocompatibility, and biostability. This study utilizes Parylene C as the piezoelectric layer, sandwiched between two copper electrodes and encapsulated with polyimide to fabricate a piezoelectric pressure sensor. Different Parylene C films of thicknesses comprising 10, 25, and 45 μm are prepared for the fabrication of the pressure sensors. The pressure sensors exhibit high sensitivities, with maximum pressure and frequency sensitivities of 87.62 and 580.95 mV Hz^−1^. Interestingly, increasing the Parylene C thickness results in an ≈300% increase in output voltage at a frequency of 9 Hz due to improved piezoelectric coefficients (*d*_33_). This study further presents a fully flexible and biocompatible Parylene C‐based dynamic pressure sensor array for integration in intelligent and smart gloves, enabling real‐time pressure monitoring and wireless data transmission using low‐range Bluetooth technology. This research should significantly advance the use of Parylene C in flexible wearable sensing technologies, with promise for applications in human–machine interfaces, healthcare, and other smart wearables.

## Graphical abstract

This study introduces a flexible, biocompatible pressure sensor using Parylene C as the active piezoelectric layer. With high sensitivity, fast response times, and wireless integration, the sensors enable real‐time pressure monitoring. The technology is demonstrated through smart gloves for healthcare and human–machine interactions, showcasing applications in wearables, robotics, and medical systems.

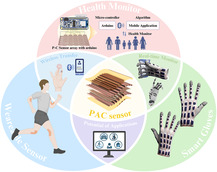

## 1 Introduction

Recently there has been a significant leap forward in the design and composition of flexible and wearable devices including pressure sensors, humidity sensors,^[^ ^1^, ^2^ ^]^ breath sensors,^[^ ^3^ ^]^ gas sensors,^[^ ^4^ ^]^ and temperature sensors^[^ ^5^ ^]^ due to advances in the healthcare systems, artificial intelligence (AI), and Internet of Things (IoT) technologies. Wearable pressure sensors have received significant interest for various applications including implantable devices,^[^ ^6^ ^]^ health monitoring,^[^ ^7^ ^]^ soft robotics,^[^ ^8^ ^]^ prosthetics,^[^ ^9^ ^]^ and biointegrated electronic devices. In principle, pressure sensors can be classified into four different types, comprising piezoresistive, capacitive, triboelectric, and piezoelectric, and have demonstrated excellent performance in terms of sensitivity and flexibility. Capacitive‐ and resistive‐type sensors generally need an external power supply like batteries for their operation, making it difficult for long‐term usage for wearable applications. To overcome this problem, exploring energy harvesting devices as self‐powered pressure sensors which can harvest the energy from environmental stimuli such as pressure is a feasible solution. However, fabrication of high‐performance self‐powered pressure sensors which can offer excellent sensitivity, suitably fast response, and recovery times, with good cyclic stability and repeatability, is a significant challenge. The triboelectric effect can be employed to produce well‐performing self‐powered sensors, though they still experience relatively high impedances, which limit how well they can interface with external readout circuitry.^[^ ^10^, ^11^ ^]^ Piezoelectric‐based pressure sensors, with tailored active material compositions to achieve high piezoelectric coefficients, are therefore attractive solutions.

Traditional pressure sensors fabricated from conventional piezoelectric materials, such as lead zirconate titanate (PZT) and barium titanate (BaTiO_3_), exhibit high piezoelectric coupling coefficients, outstanding mechanical and chemical stabilities, and high operating temperatures, meaning they exhibit higher efficiency conversions in mechanical‐to‐electrical energy and stable operation in harsh environments.^[^ ^12^, ^13^ ^]^ However their inherent rigidity with high Young's modulus (50 GPa), high density, and toxic‐containing lead considerably restrict their suitability for next‐generation wearable and implantable devices, which require flexibility, conformability, and adaptability to dynamically changing and complex surfaces.^[^ ^14^, ^15^, ^16^ ^]^ Conversely, flexible and stretchable sensors using novel polymers such as PVDF (polyvinylidene fluoride),^[^ ^17^, ^18^, ^19^ ^]^ silk,^[^ ^3^, ^20^, ^21^ ^]^ chitosan,^[^ ^22^, ^23^, ^24^ ^]^ and their composites offer numerous advantages in terms of flexibility, stretchability, and adaptability to various surfaces, opening avenues of investigation for integration with more irregular surface profiles and the human body, but exhibit low *d*_33_ values. Despite this, there are different materials which exhibit superior piezoelectricity but still lack in terms of biocompatibility, with the clear exception of biomaterials. Parylene C (PAC) is a candidate FDA (U.S. Food and Drug Administration)‐approved material which offers excellent chemical inertness, good biocompatibility, superior mechanical flexibility, high thermal stability, and suitable optical properties.^[^ ^25^ ^]^ Until now, PAC has been explored as a substrate and encapsulation material for various wearable and implantable applications,^[^ ^26^, ^27^ ^]^ but its capabilities have not been fully explored because its potential as a piezoelectric material has been largely overlooked for the fabrication of energy harvesting devices like piezo/triboelectric nanogenerators and associated applications like self‐powered pressure sensors. Recently, Sedat etal. demonstrated the use of PAC as a promising candidate for the fabrication of various types of pressure sensors using piezoelectric, piezoresistive, and capacitive elements.^[^ ^28^ ^]^ Furthermore, PAC‐based pressure sensors typically exhibit excellent sensitivity, good cyclic stability, fast responses, and recovery times, along with superior repeatability.^[^ ^29^, ^30^ ^]^

**Figure 1:**
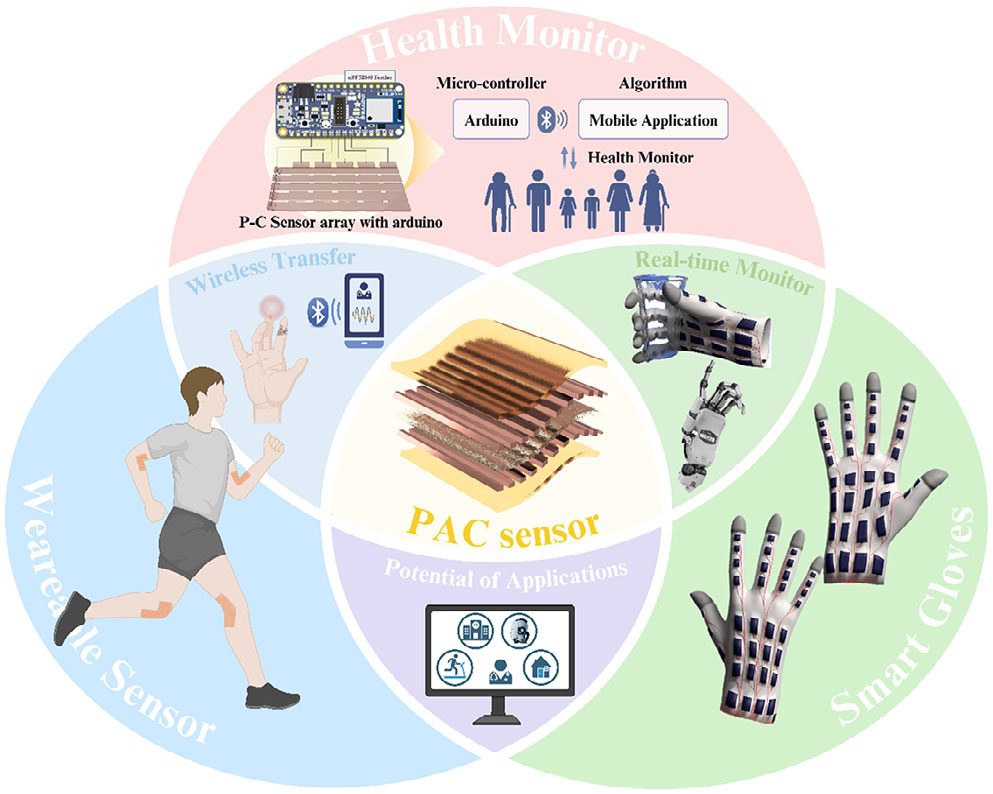
Illustration of a wireless and real‐time health monitor system including wearable sensor and smart gloves, etc. based on dynamic PAC sensor arrays and Arduino microcontroller with Bluetooth technology.

In this study, a fully flexible and biocompatible piezoelectric‐based dynamic pressure sensor array is present, which is embedded in a smart glove for real‐time pressure monitoring and low‐range wireless data transmission using Bluetooth technology, as shown in **Figure** **1**. The pressure sensor is fabricated using a simple sandwich structure with copper as the top and bottom electrodes, with PAC as the active piezoelectric layer and encapsulated with polyimide. PAC layers of different thicknesses, comprising 10, 25, and 45 μm, were prepared for the fabrication of dynamic pressure sensors. These thicknesses are postulated as feasible for practical applications. The pressure sensors exhibit maximum pressure and frequency sensitivities of 87.62 and 580.95 mV Hz^−1^ in the low‐pressure domain, ranging from 20 to 90 kPa and a frequency range of 2–9 Hz. The increase in the thickness of the PAC shows 300% increase approximately in the output voltage generated at a frequency of 9 Hz due to the significant increase in the *d*_33_. Furthermore, the sensor demonstrates a fast response time of 12 ms and recovery time of 10 ms, combined with good cyclic stability and mechanical robustness, suggesting suitability for wearable and E‐skin applications.

As a case study for demonstrator technology, intelligent and smart gloves were prepared by integrating the sensors to detect different ranges of pressure, from lightly gripping a plastic cup to fully deforming its shape. An Arduino‐based microcontroller system with a Bluetooth module is connected and the data is collected which is transferred to a personal device platform such as a mobile phone or tablet. This type of smart glove provides a significant advancement in the field of flexible and wearable sensing technologies and is postulated to be useful for and healthcare applications.

## 2 Experimental Section

### 2.1 Materials

PAC dimer was acquired from Speciality Coating Systems (SCS) and used as received. Highly conductive copper tapes (100 μm) and polyimide tapes of thickness (25 μm) were procured from RS components.

### 2.2 Preparation of PAC Film

**Figure 2:**
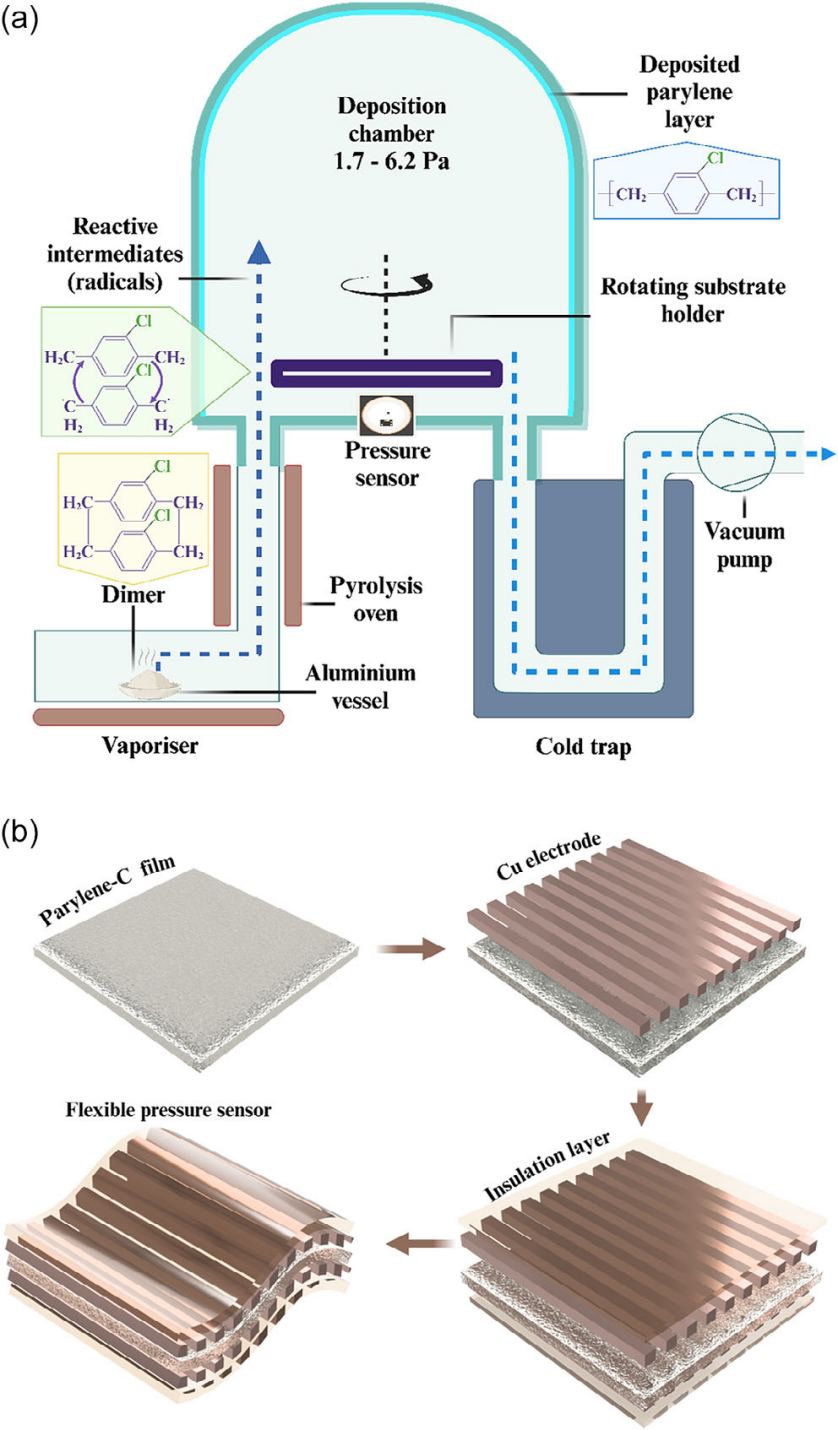
a) Schematic of the CVD process for PAC coating, showing vaporization, pyrolysis, and deposition onto a rotating substrate under low pressure (1.7–6.2 Pa); b) stepwise fabrication of the PAC sensor.

The large area, uniform, and pinhole‐free PAC film was prepared at different thicknesses using the chemical vapor deposition technique, using a SCS coating system, as shown in **Figure** **2**. In this study, deposition conditions were set to a high‐vacuum pressure ranging from 10^−^^5^ to 10^−^^6^ bar with a substrate temperature of 10 °C. Multiple thin PET (polyethylene terephthalate) sheets (15 cm × 15 cm) were placed inside the chamber and used as a carrier substrate. The substrate was cleaned using isopropyl alcohol and ultrapure deionized water to remove any unwanted residues. Next, 90 g, 50 g, and 20 g of PAC dimer powder were loaded inside the chamber (vaporizer) to obtain thicknesses of 45, 25, and 10 μm films respectively. The deposition occurred in three phases, encompassing vaporization, pyrolysis, and deposition. Initially, the dimer was added in the vaporizer and then heated up to a temperature of ≈200 °C where the dimer slowly turned from powder into the vapor with the rise in temperature and thus the vaporization phase. Next, in the pyrolysis stage, the temperature was increased up to around 680–700 °C where it transformed into a monomer. Finally, in the deposition phase a transparent film was uniformly coated on the desired substrate. The surface uniformity was tested using scanning electron microscopy (SEM), while the average surface roughness, measured by an optical 3D measurement system (Alicona), was ≈0.01 μm as illustrated in Figure S1, Supporting Information. Additionally, a relatively high crystallinity of 38.81% was achieved.

### 2.3 Fabrication of the Flexible Pressure Sensor

The flexible piezoelectric pressure sensor was fabricated using copper as the top and bottom electrodes, with the PAC as the active piezoelectric layer with an active area of the device being 2 cm × 2 cm. A simple insulator‐based capacitive structure was adopted for the piezoelectric‐based pressure sensor. A polyimide tape of thickness of 25 μm was used as an encapsulation layer. An ultrathin and long copper wire was soldered using an adhesive silver paste to use as an external contact on both top and bottom electrode. PAC films of different thicknesses of 10, 25, and 45 μm were used as the piezoelectric layers for the fabrication of the pressure sensor. Finally, a 4 × 4 pressure sensor array was fabricated and explored in the manufacture of a smart glove for touch sensor applications.

### 2.4 Characterization of the Pressure Sensor

The surface roughness and shape of the PAC films were measured using an optical 3D measurement system (Alicona Infinite Focus G4). A field‐emission SEM (FEI Nova Nano SEM) was employed to examine the surface morphology of each PAC film's nanostructure. AFM analysis was performed using Bruker systems and the analysis was performed using the nasoscope analysis software. The scanning area of 5 μm × 5 μm was considered for the surface roughness measurements, and the average surface analysis was calculated at multiple locations and an average of five regions was considered for the analysis. The crystalline structures were characterized by X‐ray diffraction (XRD, Rigaku MiniFlex 600 Diffractometer), using a diffraction angle step of 0.01° with a test range of 10°–70°. Chemical structures were analyzed by Raman spectroscopy LabRAM HR system, manufactured by Horiba Jobin Yvon and equipped with the Ventus 532 laser system, 100 mW, 532 nm). Fourier‐transform infrared spectroscopy (FTIR, Bruker Platinum A225) was utilized to examine the PAC film's chemical composition and functional groups. The thickness of the PAC films was measured by a gauge (Logitech Limited Measuring Gauge CG10), and the sensitivity of the pressure sensors was calculated using the TIRA vibration system which can generate periodically compressive loads on the sensors (TV 50 018, TIRA GmbH) as shown in Figure S2, Supporting Information. The output voltage signal under periodic deformation was recorded by a digital storage oscilloscope (DSOX3014T). Additionally, long‐term cyclic pressure was applied to evaluate the stability of the sensors and monitored by the oscilloscope.

### 2.5 Statistical Methodology

The raw data obtained from the experiments were analyzed using the following processing methods to improve the reliability and reproducibility of the results. Ten samples were produced for each of the PAC sensors, with thicknesses of 10, 25, and 45 μm. The evaluation metrics for each thickness of sensor were derived from the mean value of the obtained data from the respective ten samples. The sensitivity of each sensor to pressure or frequency was determined from the ratio of the change in the output response voltage to the applied input pressure or frequency. However, the relationship between voltage and pressure or frequency was not completely linear. So, the experimental discrete data points were fitted using the least squares method in OriginLab software, where the slope and variance (*R* ^2^) were calculated to quantify the deviation between the fitted line and the actual values as shown in Figure S3, Supporting Information. This approach ensures a more accurate assessment of the data.^[^ ^31^ ^]^ The output voltage *y* can be expressed by the linear Equation (1)

(1) y=Sx+b 

where *S* represents the sensitivity (the slope of the fitted line), *x* is the applied pressure or frequency, and *b* denotes the intercept. The coefficient of determination *R* ^2^ for evaluating the fit quality is defined as Equation (2)

(2) $$R^{2}=\frac{1}{n}\sum_{i=1}^{n}{\left(y_{i}-{\hat{y}}_{i}\;\right)}^{2}$$

where *n* is the number of data points, $y_{i}$ is the measured output voltage, and ${\hat{y}}_{i}\;$ is the corresponding predicted voltage obtained from the linear fit.

## 3 Results and Discussion

### 3.1 Material Characterization

**Figure 3:**
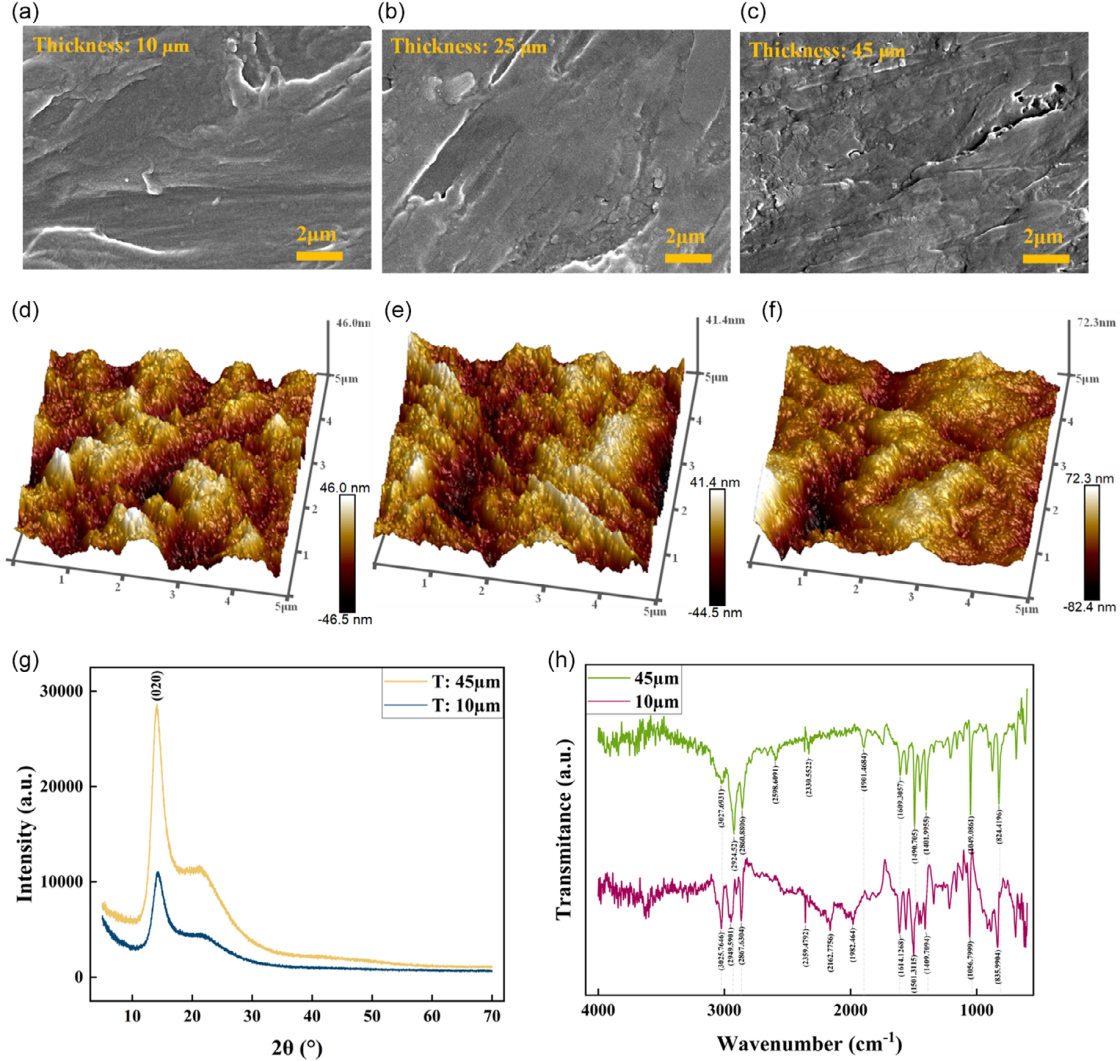
SEM images of PAC film surfaces with thicknesses of a) 10 μm, b) 25 μm, and c) 45 μm (scale bar: 2 μm), and corresponding AFM phase images (scan area: 5 μm × 5 μm) with thicknesses of d) 10 μm, e) 25 μm, and f) 45 μm. g) XRD plot and h) FTIR for the thickness 10 and 45 μm PAC films.

To understand the structural, chemical, and morphological characteristics of the material, various material characterization techniques including SEM, XRD, and FTIR were performed on the PAC films as represented in **Figure** **3**. SEM was utilized to examine the surface morphological changes in the PAC films, providing detailed insights into their microstructural features. As shown in Figure 3a–c, the SEM images of different thickness PAC films exhibited mild surface roughness and slight morphological variations, rather than a completely smooth. All PAC films deposited under similar conditions using chemical vapor deposition (CVD) with thicknesses of 10, 25, and 45 μm revealed incremental changes in surface textures with increasing film thickness and gradually developed more obvious morphological features indicative of increasing crystallinity at thicker films. There are no significant defects, such as cracks or pinholes were observed, indicating consistent structural integrity across all films. Additionally, as shown in Figure 3a, there are no clearly defined grain boundaries in the thinner PAC films, confirming their amorphous nature at lower thicknesses. In contrast, crystalline‐like structures appear in thicker PAC films, as illustrated in Figure 3c, suggesting a gradual increase in crystallinity with increasing film thickness. To further clarify the quantitative details of the observed surface morphology, AFM analysis was performed on PAC films with thicknesses of 10, 25, and 45 μm. The 3D Atomic Force Microscopy (AFM) surface topography images shown in Figure 3d–f showed the existence of clear nanoscale surface features. The measured arithmetic surface roughness (Ra) values were ≈11.95 nm for the 10 μm film, 10.72 nm for the 25 μm film, and 11.09 nm for the 45 μm film. These AFM results indicate consistently moderate surface roughness, with Ra values narrowly ranging from ≈10 to 12 nm across all film thicknesses. Minor variations in Ra values observed among the PAC films may be attributed to CVD process‐induced internal stresses and changes in polymer molecular chain alignment, which is consistent with previous studies on PAC thin films deposited under similar conditions.^[^ ^32^, ^33^ ^]^ Consequently, this quantitative AFM analysis strongly supports the SEM observations, confirming the structural uniformity and nanoscale surface consistency of the PAC films. Next, XRD was used to provide insights into its amorphous, semicrystalline, crystallinity nature, and molecular arrangement of the PAC films which largely vary depending on processing conditions and post treatment methods. Here, XRD was performed on the PAC films with thicknesses of 10 μm and 45 μm deposited under similar conditions using CVD. Figure 3g shows the XRD spectra of the PAC films (10 and 45 μm) for a 2q angle range varying from 1°–70° with a step size of 0.05°. It is observed that a single sharp diffraction peak centered at a 2q angle of ≈15° corresponds to the (020) plane with the monoclinic crystal unit cell, correlating to the a phase with a semicrystalline nature.^[^ ^34^ ^]^ The obtained results suggest that the peak intensity of the thicker film is higher than that of the thinner film, due to the variation in the crystallinity of the film from the surface confinement of the layers inside the PAC. To further understand the crystallinity of the film with the variation in thickness, the degree of crystallinity (*X*_c_) was calculated using Equation (3).

(3) $$X_{c}=\frac{A_{c}}{A_{c}+A_{A}}\times 100\left(\%\right)$$

Here, *A*_c_ and *A*_A_ are the areas under the curve for the crystallinity and amorphous peaks respectively, which were determined using curve fitting using OriginPro (OriginLab Corporation). The calculated *X*_c_ values were obtained as 36.45% and 38.81% for the 10 μm‐and 45 μm‐thick PAC films respectively, which clearly indicate that the rise in thickness results in the increasingly crystalline nature of the film due to the higher level of molecular packaging and where the interactions such as van der Waals forces and hydrogen bonding and polymer chains align to form a regular crystal region within the material.^[^ ^35^ ^]^ The full width at half maximum (FWHM) is one further important parameter in quantifying the crystallinity of the PAC films, where it is inversely proportional to the crystal or grain size. The FWHM values of both the 10 and 45 μm‐thick films were calculated using the Scherrer equation and were therefore determined to be 18.19° and 14.5° respectively, with the corresponding grain sizes as 0.44 and 0.55 nm respectively. It is clear from the results that the increase in the thickness of the film produces an equivalent rise in the grain size and in the overall crystallinity of the film, which is consistent with the literature.^[^ ^36^ ^]^ Examination through Raman spectroscopy confirmed the characteristic Raman bands of PAC films with different thicknesses, as shown in Figure S4, Supporting Information. Representative Raman spectra of PAC films with thicknesses of 10, 25, and 45 μm are presented in Figure S4a, Supporting Information. Clearly, all spectra exhibit well‐defined characteristic peaks, verifying the successful deposition of PAC. Specifically, the prominent peaks appearing at ≈689, 1007, 1339, and 2930 cm^−^^1^ are attributed to C–Cl stretching vibrations, benzene ring symmetric breathing vibrations, C–H bending vibrations, and C–H stretching vibrations, respectively.^[^ ^37^, ^38^ ^]^ Thus, these peaks generally confirm the crystalline characteristics of PAC. Raman spectra obtained from PAC films with varying thicknesses revealed significant increases in peak intensities corresponding to increased film thickness, as illustrated in Figure S4b–e, Supporting Information. Particularly, the Raman intensity at the 1339 cm^−^^1^ peak notably increased from ≈9590 for the 10 μm film to about 18 006 for the 25 μm film and further to around 30 061 for the 45 μm film. This pronounced increase in Raman intensity directly corroborates the enhanced crystallinity identified by XRD analysis for thicker PAC films.^[^ ^39^ ^]^ Furthermore, minor shifts in Raman peak positions were detected, which could originate from internal stresses and changes in molecular orientation resulting from variations in film thickness.^[^ ^39^ ^]^ Collectively, these Raman results clearly demonstrate that an increase in film thickness results in enhanced crystallinity of PAC layers. To gain deeper insights into the chemical composition and functional groups within the PAC, FTIR analysis was performed on both 10 μm and 45 μm‐thick films. The results in Figure 3h show the transmittance spectrum for both PAC films across a spectral range of 100–4000 cm^−1^. The FTIR typically exhibits the spectral peaks for 10 μm‐thick PAC films at wave numbers of (835, 1056, 1409, 1501, 1614, 1982, 2162, 2359, 2867, 2949, and 3025) cm^−1^ respectively, where these were observed at (824, 1049, 1401, 1490, 1609, 1901, 2330, 2598, 2860, 2924, and 3027) cm^−1^ for 45 μm‐thick PAC films respectively. The shifts of spectral peaks could be due to alterations in intermolecular forces such as van der Waals interactions or hydrogen bonding. Furthermore, strain or stress within the thicker PAC film during the fabrication process such as drying might slightly modify bond lengths or angles, thereby leading to minor shifts in the observed peak positions. The spectral peaks from 1409 to 1614 cm^−1^ relate to the in‐plane vibrations of the benzene ring, as well as the stretching vibrations of the C—C and C=C present in the para‐xylene monomer. The peak at 1056 cm^−1^ corresponds to the C—Cl bond which indicates the presence of the chlorine in the monomer. Similarly, the peaks in between 2800 and 3025 cm^−1^ correspond to the C—H bond on the atomic ring which matches well with the literature.^[^ ^40^ ^]^

### 3.2 Electromechanical Characterization

**Figure 4:**
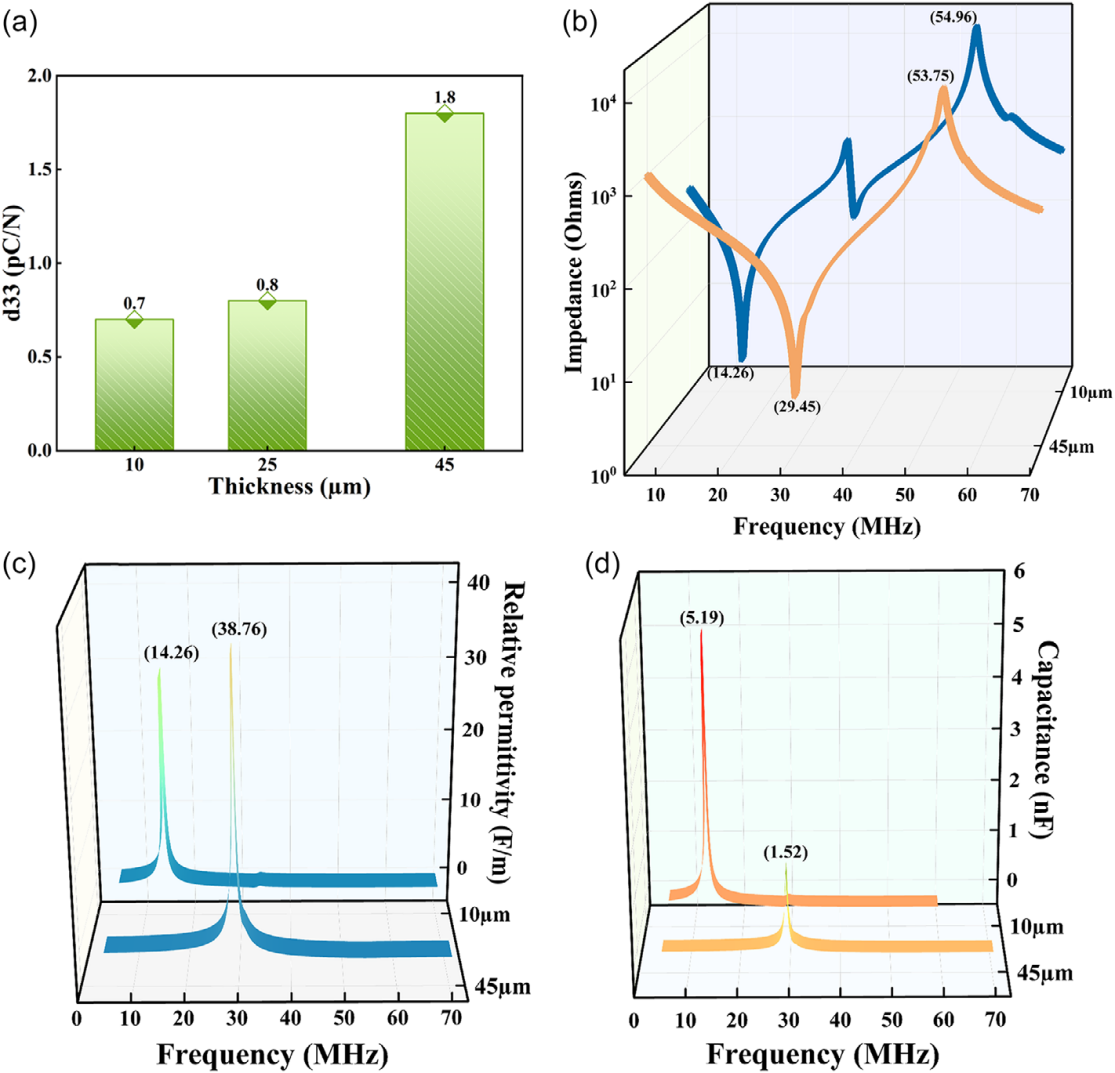
a) The variation of piezoelectric coefficient (*d*_33_) of PAC under different thicknesses of 10, 25, and 45 μm. b) Impedance analysis of the PAC films of thicknesses comprising 10 μm (orange) and 45 μm (blue). c) and d) Relative permittivity and specific capacitance values of the capacitive structured films of 10 and 45 μm.

The *d*_33_ measurements were performed to determine the piezoelectric characteristics of the prepared PAC films, to quantify the material's ability to generate an electrical charge in response to external mechanical pressure. **Figure** **4**a shows that the *d*_33_ measurement values were 0.7 pC/N at 10 μm thickness, 0.8 pC/N at 25 μm, and 1.8 pC/N at 45 μm. The increase in *d*_33_ with film thickness indicates an enhancement in the piezoelectric properties with increasing magnitudes of film thickness. This enhancement can be attributed to the improved crystallinity and alignment of polymer chains in thicker films, which facilitates better dipole alignment and charge generation under mechanical stress. Dielectric properties of the piezoelectric film determine the capacitance of the material and play a crucial role in understanding the performance and functionality of the piezoelectric films. To evaluate the dielectric properties of the PAC films, impedance measurements were performed on PAC films of 10 and 45 μm in thickness, sandwiched between the Au electrodes deposited via E‐beam evaporation. Further, the impedance of the film increases with increasing thickness, and for the capacitance, as the thickness increases, the capacitance decreases. For a few microns of film thickness, the dielectric constant remains stable with slight fluctuations. For example, Coelho et al. showed that the capacitance of PAC films decreased from 9.48 nF cm^−2^ to 1.83 nF cm^−2^ as the film thickness increased from 0.24 to 1.45 μm; however, the relative permittivity was 2.6 when the thickness is 0.24 μm. For films with thickness from 0.46 μm to 1.45 μm, the relative dielectric constants are stable in the range of 3.24–3.20. Thus, including an intermediate thickness (25 μm) would not substantially alter the trend or provide additional insight beyond what is already inferred from the end points.^[^ ^29^ ^]^ Figure 4b shows the variation of the electrical impedance with respect to frequency ranging from 1 MHz to 70 MHz. It is observed that the piezoelectric coupled resonance peak was centered around 14.26 MHz for the 10 μm film and at 29.45 MHz for the 45 μm film, where the antiresonance peaks were measured at 54.96 MHz and 53.75 MHz for the 10 μm and 45 μm films, respectively. These resonance peaks are useful in the generation of microvibrations for various devices like ultrasonic sensors and actuators, because they allow for the tuning of device operating frequencies to achieve optimal performance in specific applications. The relative permittivity (*ε*_r_) or dielectric constant and capacitance (*C*) of the PAC films were calculated using Equation (4) and (5) as shown below.

(4) $$\left|X_{c}\right|=\frac{1}{2\pi fC}$$

(5) $$C=\frac{\varepsilon A}{d}=\frac{\varepsilon_{0}\varepsilon_{r}A}{d}$$

Here, *A* is area of the device (2.5 cm^−2^), *ε*_0_ is the vacuum permittivity (8.85 × 10^−12 ^F m^−1^), *d* is the thickness of the PAC (10 and 45 μm), and *f* is the applied frequency (MHz). The capacitance values are obtained as 5 nF and 1.5 nF for 10 μm and 45 μm, respectively, as shown in Figure 4c,d. The results suggest that the thinner film exhibits higher *C* as compared to the thicker film due to the inverse relationship between capacitance and thickness. Similarly, the relative permittivity (*ε*_r_) of the device was obtained as 27.89 at 14.26 MHz and 31.76 at 29.45 MHz, for 10 μm and 45 μm‐thick films respectively. This increase in relative permittivity with thickness may be attributed to enhanced polarization due to a greater volume of dipolar entities in the thicker film, indicating that film thickness can influence the dielectric response of PAC films.

**Figure 5:**
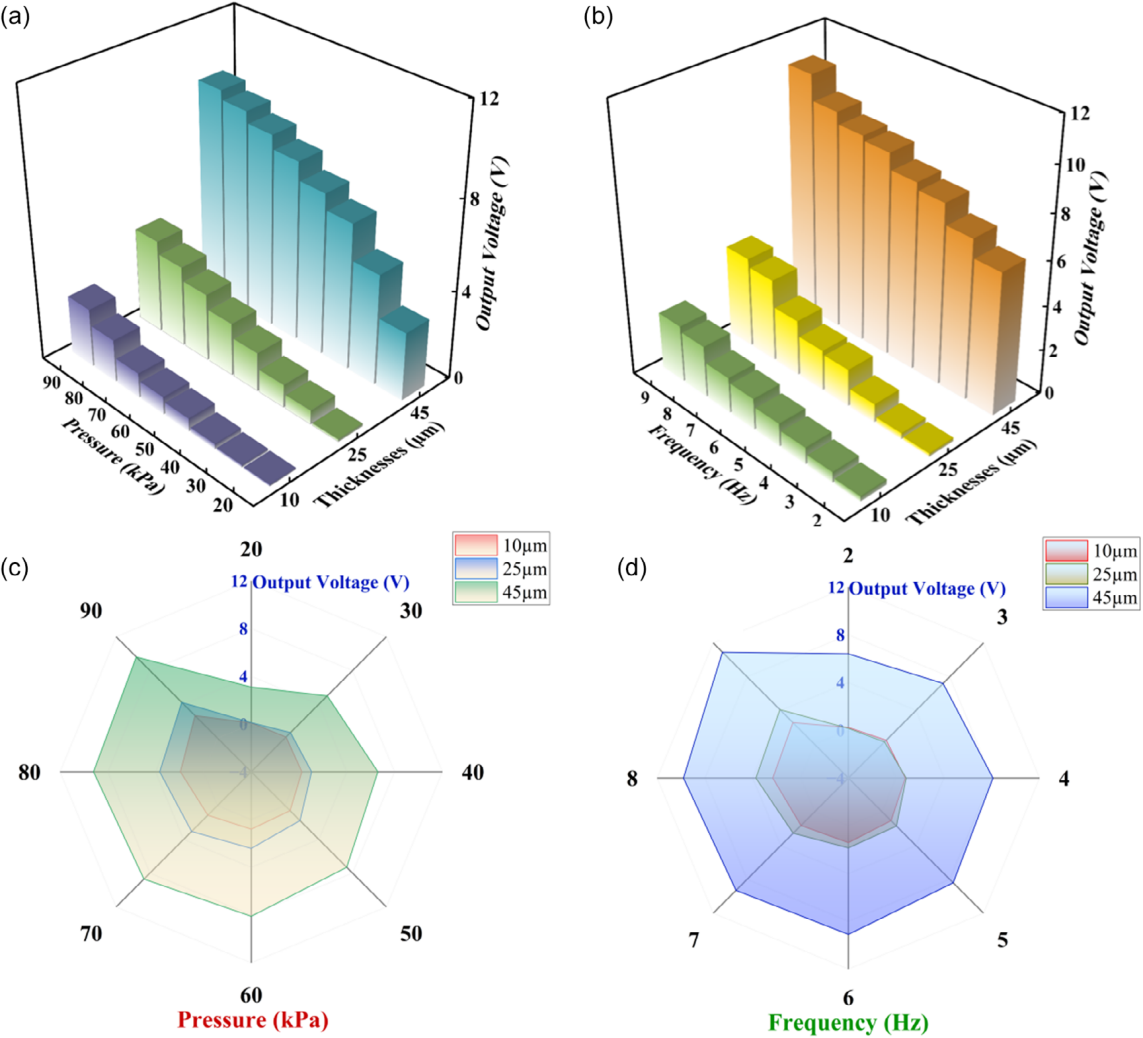
Changes in output voltage for PAC films under varying conditions; a,b) The output voltage variations with respect to applied pressure and frequency, respectively, for PAC films with different thicknesses and output response voltages with increased c) pressure and d) frequency for PAC films with thicknesses of 10, 25, and 45 μm.

To evaluate the performance of PAC‐based dynamic pressure sensors, a custom‐made setup with a mechanical shaker, linear motor, source meter, and commercial force calibration sensor was integrated to the system as shown in **Figure** **5**. The copper‐based lead lines were extended and connected to the mixed‐signal oscilloscope for capturing the output waveform, and a robust grounding was ensured to mitigate unwanted electromagnetic fields. The shaker can generate controlled mechanical vibrations varying from 0.25 N to 32 N with a frequency range from 1 to 10 Hz with minimal distortions. In order to generate the required loading/unloading motion to produce a periodic stress on the pressure sensor, the linear motor is vibrated vertically. The applied stress on the piezoelectric film produces a piezoelectric potential that generates a charge accumulated at the electrodes and as the applied stress is removed then the electrons will move back in the opposite direction resulting in removal of the piezoelectric potential.

**Figure 6:**
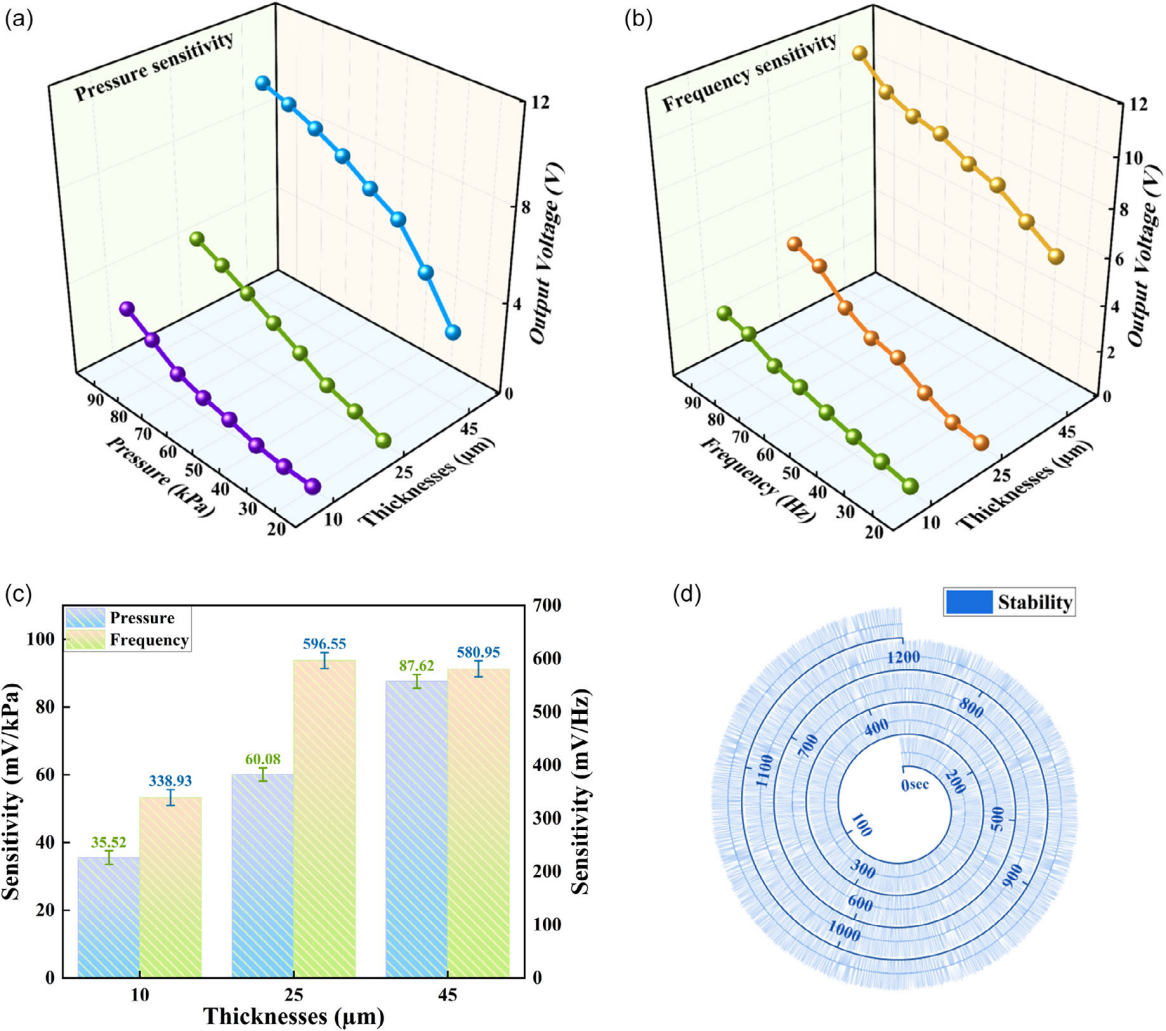
Sensitivity plot for a) pressure and b) frequency with output voltage changes, showing linear increases in pressure and frequency, respectively, for PAC films with different thicknesses. c) Comparison of pressure and frequency sensitivities for PAC sensors with different thicknesses and d) cyclic stability and over 1200 s for 45 μm thickness PAC films.

To verify the piezoelectric nature and sensitivity of the pressure sensor fabricated with PAC‐based films of thicknesses of 10, 25, and 45 μm, different electromechanical tests were performed with various forces at multiple frequencies. A pressure increment test was first conducted at a fixed frequency of 8 Hz, as illustrated in Figure 5a. The applied pressure was increased in increments of 10 kPa, across a range of 20–90 kPa. The output voltage from all samples demonstrated a significant upward trend with the increase of pressure for all the sensors of different thickness. More specifically, as the pressure increases from 20 to 90 kPa, the output voltage rises from 0.09 to 2.65 V, from 0.12 to 4.22 V, and from 3.1 V to 9.6 V, respectively, corresponding to PAC films with thicknesses of 10, 25, and 45 μm. Similarly, Figure 5b shows the results of a frequency increment test where the frequency was incremented in steps of 1 Hz, from 2 to 9 Hz under a fixed pressure of 90 kPa. The output voltage exhibits a distinct upward trend as the frequency increases when applied to films of all thicknesses studied. As the frequency of the applied pressure ascends from 2 to 9 Hz, the output voltage rises from 0.24 to 2.57 V, from 0.016 to 4.1 V, and from 6.4 to 10.9 V, corresponding to PAC films with thicknesses of 10, 25, and 45 μm respectively. As demonstrated through the results shown in Figure 5c, although the output voltage increases significantly with the increase of film thickness, thicker films have a greater response on high pressure. For example, when the thickness of the film is increased from 10 to 25 μm, the output voltage only marginally increases from 0.09 to 0.12 V at 20 kPa, but for higher applied pressures of 90 kPa, the output voltage experiences a significant increase from 2.68 to 4.22 V. Figure 5d shows that with the augmentation of the thickness of the PAC film, the output voltage generally undergoes a significant elevation at the same frequency, except in the low‐frequency scenario. When a pressure of 2 Hz is applied, for film thicknesses ranging from 10 to 25 μm and ultimately to 45 μm, the corresponding output voltages are 0.024, 0.016, and 6.4 V. It can be discerned that films of 10 and 25 μm manifest minimal responses to low‐frequency pressure. In contrast, the 45 μm film exhibits a remarkably high output voltage. However, in a relatively higher‐frequency range, such as 9 Hz, the output voltages corresponding to film thicknesses of 10, 25, and 45 μm are 2.57, 4.1, and 10.9 V respectively. Compared to the voltage changes at low frequencies, this is highly conspicuous. This can be accounted for by the fact that films of 10 and 25 μm exhibit smaller deformations under low‐frequency pressures when compared to the 45 μm film. By observing the output characteristics of different film thicknesses under a range of applied pressures and frequencies, a pressure sensor fabricated with a relatively thick PAC film can reliably and effectively detect both pressure and frequency simultaneously, due to the relatively high sensitivity to both pressure variations and frequency changes, presenting significant academic and technological value. In addition, response time, another important performance indicator for sensors, is also tested. Response time is defined as the duration for which the output voltage increases from the baseline to the maximum value when pressure is applied to the sensor. As indicated in Supporting Information, shown in Figure S5–S7, the response time for the 45 μm PAC‐based sensor is ≈12 ms. Conversely, the recovery time is the duration for the output voltage when reverting from the maximum value to the baseline value upon the removal of force from the sensor, with an approximate measurement of 10 ms. Sensitivity (*S*) is a key performance metric of the fabricated PAC‐based pressure sensor. The sensitivity of each sensor to pressure or frequency was determined through Equation (1) and Equation (2). *S* of the pressure sensor was calculated from the slope of the plot, as shown in **Figure** **6**a,b, which exhibits generally linear behavior or a linear rise in the output voltage with similar increase trends in the pressure and frequency. Based on these data, *S*_p_ and *S*_f_ were calculated in accordance with the linear fitting equation, as shown in Figure S3, Supporting Information. Figure 6c shows the sensitivity of the sensor under different pressures and frequencies varying from low to high regimes, in terms of 20 kPa–90 kPa at a constant frequency of 8 Hz and 2 Hz–9 Hz at a constant pressure of 90 kPa, respectively. As depicted in Figure 6c, the value of *S*_p_ rises with the increase in the PAC thickness, being 35.52, 60.08, and 87.62 mV kPa^−1^, corresponding to thicknesses of 10, 25, and 45 μm respectively. However, for the frequency response (*S*_f_), as the film thickness gets thicker from 10 μm to 45 μm, *S*_f_ experiences a large increase followed by a slight decrease. More specifically, *S*_f_ can be measured as 338.93, 596.55, and 580.95 mV Hz^−1^ respectively, for the 10, 25, and 45 μm PAC thicknesses. The results suggest that *S*_p_ is significantly enhanced by the increase in the thickness of PAC, demonstrating its potential in applications for fabricating a customized dynamic pressure sensor with specific values of *S*_p_ that are appropriate for precise detection of frequency and pressure. The obtained results also demonstrate that the device exhibits stability, is noise free, fast, and capable of repeatable output voltages under different pressures and frequencies, clearly suggesting its suitability for applications relating to wearable and implantable pressure sensing. Finally, to acquire a deeper understanding of the stability and reliability of the PAC pressure sensor, a more sustained cycling test was conducted. With more than 1200 cycles, as shown in Figure 6d, the results show that the device has excellent cycling stability with almost no deviation in output voltage. In order to further verify the long‐term stability and reliability of the pressure sensor, 10‐day continuous fatigue tests on sensors of various thicknesses were conducted. Specifically, the sensors were subjected to a pressure of 90 kPa at 5 Hz with a vibrometer at room temperature of 23–25 °C and a humidity of 30%–35%. The tests were conducted continuously for a period of 4 h on each day, and the output voltage values were recorded. The results of this study are illustrated in Figure S8, Supporting Information, which shows the average output voltage values for 10, 25, and 45 μm thickness sensors over a 10‐day period. It is evident that the sensors demonstrate consistent performance over a long period of time, exhibiting minimal standard deviation. For example, for the 45 μm‐thick sensor, the voltage output fluctuated between 9.1 and 9.5 V over the 10‐day period, with a mean voltage of 9.37 V and a standard deviation of 0.1889 V, demonstrating that the PAC exhibits long‐term stability and reliability in its piezoelectric behavior. Hence, the stability and durability of the sensor could meet actual application scenarios to realize continuous, accurate detection over long periods of time.

### 3.3 Application in Wearable Systems

**Figure 7:**
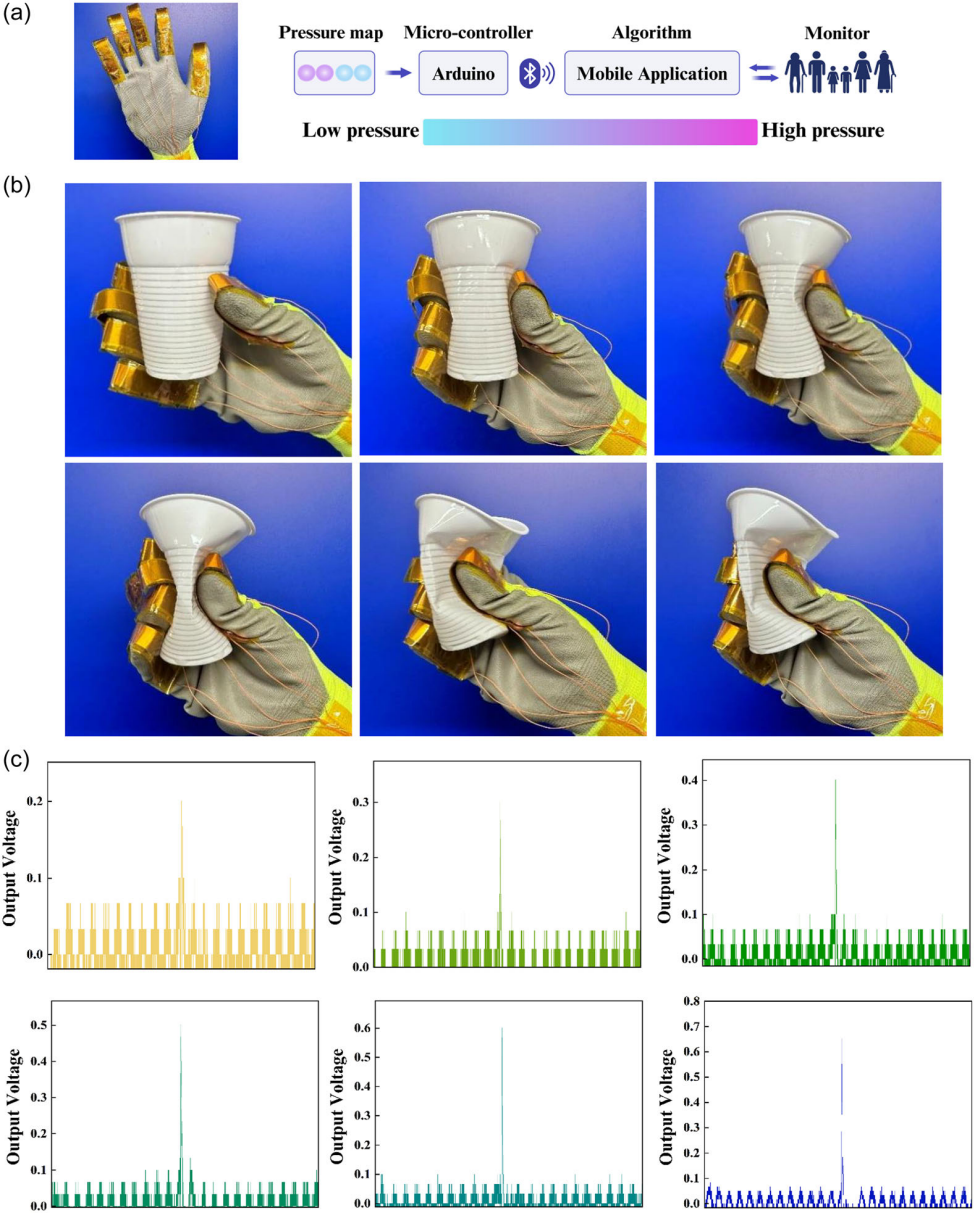
a) Smart glove embedded with PAC‐based piezoelectric sensors for real‐time grip monitoring and data transmission via an Arduino to a mobile application; b) the smart gloves exhibiting the ability to monitor real‐time object interactions; and c) the real‐time output voltage signals recorded for each movement.

Human–machine interactions are gaining significant attention owing to accelerating IoT, soft robotics, medical devices, machine learning, and AI technologies. To successfully implement these applications, various sensors, including pressure sensors that offer high performance and mechanical flexibility, are vital. To demonstrate such an application here, the fabricated flexible and self‐powered pressure sensors were integrated into a textile‐based smart glove, as shown in **Figure** **7**a. A Nordic nRF52840 System‐on‐Chip (SoC) was employed due to its low power consumption and integrated Bluetooth low energy (BLE) capabilities to realize wireless and real‐time monitor of a five‐channel sensor array embedded in the smart gloves as demonstrated in Figure S9, Supporting Information. The SoC is equipped with a 12‐bit analogue‐to‐digital converter that facilitates the digitization of analogue voltage outputs from the piezoelectric sensor array. Additionally, it supports BLE data rates of up to 2 Mbps, a capability that renders it well suited for applications necessitating rapid data exchange. The transmission of data and the development of firmware were both conducted within the Arduino IDE environment. The digitized sensor outputs were then transmitted over the serial peripheral interface and visualized through a mobile application.^[^ ^31^ ^]^ As demonstrated in Figure 7b, a series of experiments were performed to validate the glove's ability to monitor real‐time object interactions. In the demonstration, the smart glove is used to interact with an object; in this study, a cup, where the real‐time output voltage signals are recorded for each movement, indicates the varying pressure exerted on the object by the fingers. Real‐time monitoring data, in terms of output voltage, is shown in Figure 7c, corresponding to the key motional interactions as depicted in Figure 7b. The output voltage gradually increases from 0.2 to 0.7 V as the grip pressure increases. This incremental rise in voltage reflects the glove's ability to accurately detect and quantify subtle changes in applied pressure. Additionally, this application demonstrates the practical and effective potential of PAC‐based pressure sensors in various domains including wearable technology and healthcare, robotics, and human–computer interaction due to their ability to accurately monitor and transmit grip pressure data in real time.

**Table** **1** presents a comparison of the performance of polymer‐based pressure sensors in the three dimensions of sensitivity, response time, and biocompatibility. It is evident from the table that pressure sensors manufactured with 45 μm‐thick PAC films in this study demonstrate remarkable sensitivity and biocompatibility, as well as a relatively fast response time of 12 ms. The polymeric materials PVDF‐TrFE (polyvinylidene fluoride‐trifluoroethylene), PVDF, gelatin, chitosan, PLLA‐Glycine, and PAC employed in this study all possess outstanding biocompatibilities and are anticipated to have extensive prospects in biomedical applications. However, the PAC sensitivity is the highest, with chitosan and PVDF‐TrFE exhibiting the lowest sensitivities of 2.82 and 2.5 mV kPa^−1^, respectively. These sensitivities are challenging to meet in practical applications. Gelatin demonstrates optimal performance at 37.9 mV kPa^−1^; however, this is considerably lower than that of the PAC, which exhibits a value of 87.62 mV kPa^−1^ in this study. For response time, although PVDF‐TrFE demonstrates a relatively rapid response time of 5 ms, its sensitivity is significantly lower than that of PAC. In comparison to the majority of pressure sensors, the PAC demonstrates a significantly reduced response time of 12 ms, which is considerably less than the 193 ms exhibited by gelatin. Additionally, although pressure sensors based on the polydopamine @ PZT/polyimide composite exhibits high sensitivity, there is no data to indicate that the composite with PZT doping is biocompatible. In brief, the pressure sensor fabricated from PAC features high sensitivity, a short recovery response time, and biocompatibility, which can fulfil the requirements of pressure detection in the medical field.

**Table 1:** State‐of‐the‐art comparison of the polymer‐based PENG devices.

Material	Sensitivity	Response time	Biocompatibility	Ref
PVDF‐TrFE	2.5 mV kPa^−1^	5 ms	YES	[41, 42]
PVDF	27 mV kPa^−1^ at 20 kPa	–	YES	[43]
PVDF‐ZnO	33 mV kPa^−1^	16 ms	YES	[44]
Gelatin	37.9 mV kPa^−1^	193 ms	YES	[45]
Polydopamine @ PZT/ polyimide composite	240 mV N^−1^	150 ms	NO	[46]
Chitosan/β‐glycine	2.82 mV kPa^−1^	–	YES	[47]
PLLA‐Glycine	13.2 mV kPa^−1^	10 ms	YES	[48]
Parylene C @ 45 μm thicknesses	87.62 mV kPa^−1^	12 ms	YES	This work

## 4 Conclusion

In summary, the proposed dynamic piezoelectric‐based pressure sensor array using PAC film provides excellent pressure and frequency sensitivities with mechanical flexibility and biocompatibility. Various material characterization processes like SEM, AFM, XRD, Raman, FTIR, and *d*_33_ measurements were performed to analyze the morphological and structural properties of PAC. Various optimization studies of different PAC film thicknesses were used to enhance the piezoelectric coefficient, thereby improving the output voltage performance, resulting in the higher sensitivities that were successfully validated in this study. Furthermore, the sensors exhibit fast response and recovery times, with a level of cyclic stability demonstrating its suitability for high‐speed flexible and wearable electronic applications including human healthcare monitoring systems, human–machine interfaces, and E‐skin. Finally, an intelligent and smart glove with wireless data transfer capability using Bluetooth technology was successfully demonstrated by integrating a PAC‐based pressure sensor array for real‐time pressure monitoring, postulated for use in applications such as knee replacement surgeries or physiological monitoring of human motions. This work will lay a strong foundation for the development next‐generation wearable and implantable sensing technologies that bridge the gap between the human and machines, empowering advanced networking, communication, and monitoring systems.

## Conflict of Interest

The authors declare no conflict of interest.

## Author Contributions

**Zhao Wang**: conceptualization (lead); data curation (lead); formal analysis (lead); investigation (lead); methodology (lead); validation (lead); visualization (lead); writing—original draft (lead). **Bhavani P. Yalagala**: conceptualization (lead); data curation (lead); formal analysis (lead); investigation (lead); methodology (lead); validation (lead); visualization (lead); writing—original draft (lead). **Hadi Heidari**: funding acquisition (supporting); project administration (supporting); supervision (supporting). **Andrew Feeney**: funding acquisition (lead); project administration (lead); supervision (lead); writing—review and editing (equal).

## Supporting information

Supplementary Material

## Data Availability

The data that support the findings of this study are available from the corresponding author upon reasonable request.
