# Supplementary material for: Highly Sensitive Parylene C‐Based Flexible Pressure Sensors for Wearable Systems
Source: Small Sci. 2025 May 14;5(7):2500081. doi: 10.1002/smsc.202500081 (PMC12257884; doi:10.1002/smsc.202500081)
Supplement: Supplementary file 1 — Supplementary Material [file SMSC-5-2500081-s001.pdf]

Supporting Information

on

Highly Sensitive Parylene C Based Flexible Pressure Sensors for Wearable Systems

Zhao Wang[1], *Bhavani P. Yalagala*[2], *Hadi Heidari*[1,2], *and Andrew Feeney*[1, *]

[1] Centre for Medical and Industrial Ultrasonics (C-MIU), James Watt School of Engineering, University of Glasgow, Glasgow, G12 8QQ, U.K

[2] Microelectronics Lab (meLAB) Group, James Watt School of Engineering, University of Glasgow, Glasgow, G12 8QQ, U.K

E-mail: Andrew.Feeney@glasgow.ac.uk

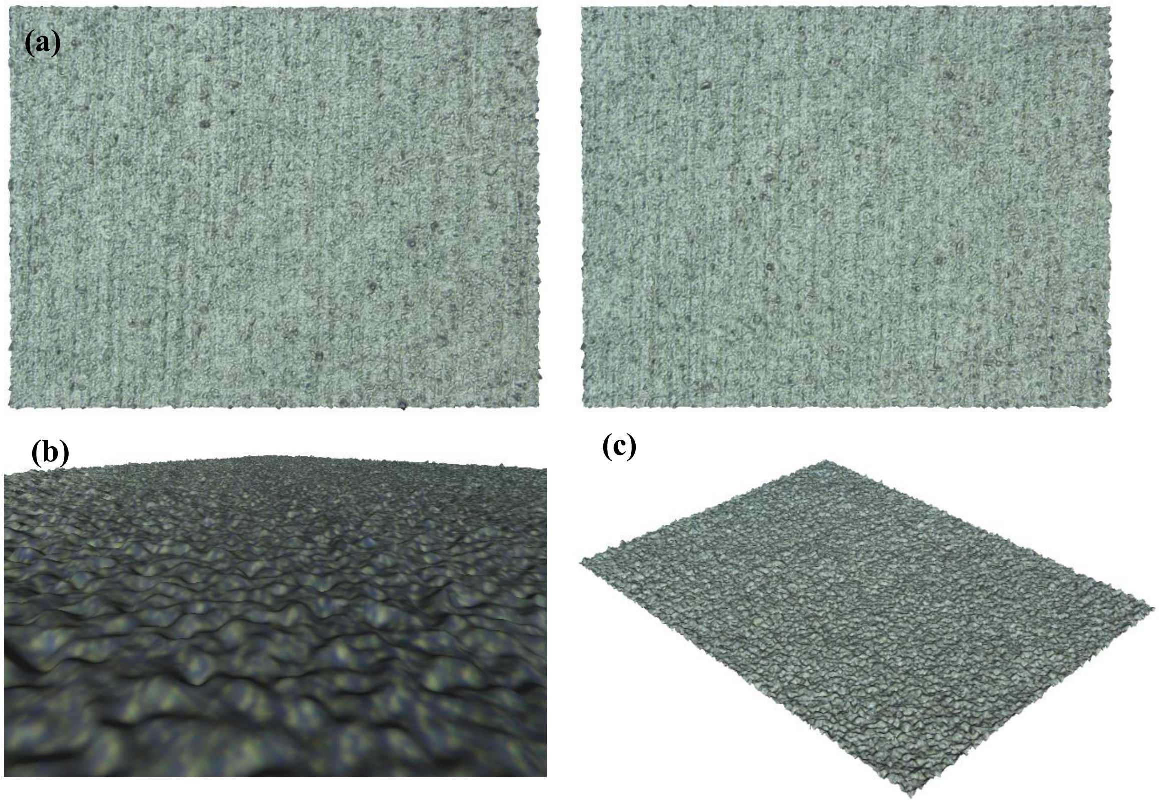

Figure S1. Images of PAC films surface (a) morphology and (b), (c) roughness and texture measured by an optical 3D measurement system.

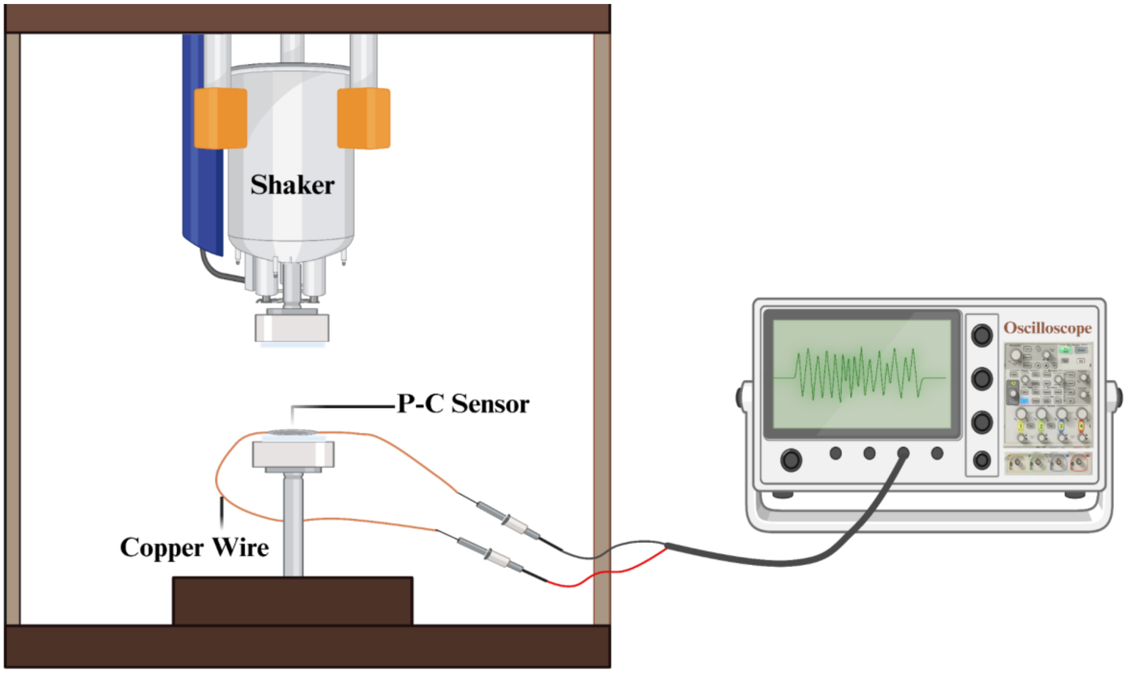

Figure S2. Schematic of the vertical vibration system setup, which serves as a tunable external force application system with adjustable pressure and frequency, designed for the performance characterization of PAC sensors.

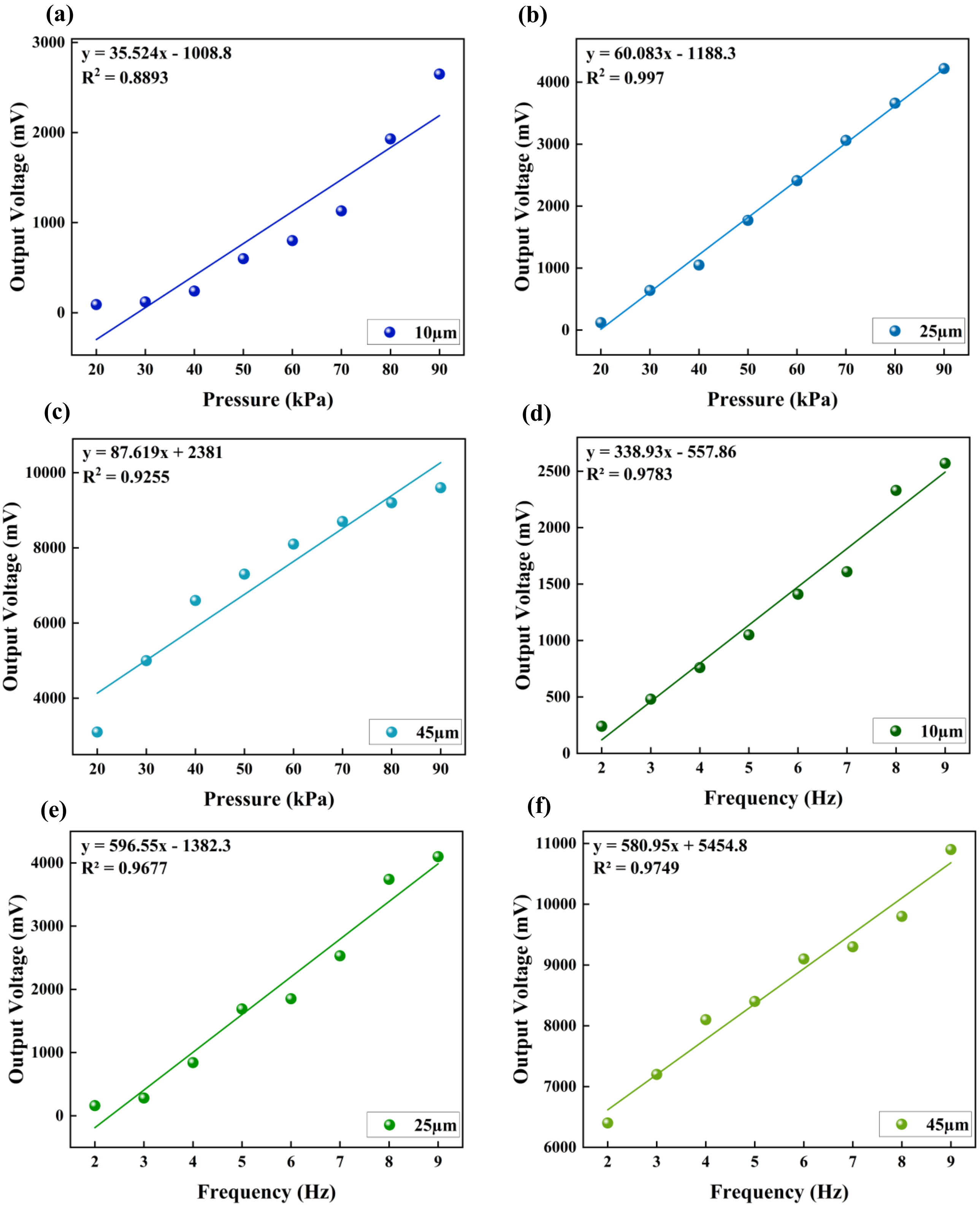

Figure S3. (a–c) Output voltage responses as functions of applied pressure for sensors with thicknesses of **(a)** 10 μm, **(b)** 25 μm, and **(c)** 45 μm, corresponding to pressure sensitivities of 35.52 mV/kPa, 60.08 mV/kPa, and 87.62 mV/kPa, respectively. **(d–f)** Output voltage responses as functions of frequency for sensors with thicknesses of **(d)** 10 μm, **(e)** 25 μm, and **(f)** 45 μm, corresponding to frequency sensitivities of 338.93 mV/Hz, 596.55 mV/Hz, and 580.95 mV/Hz, respectively. Linear fitting equations and the corresponding R^2 values are provided in each plot.

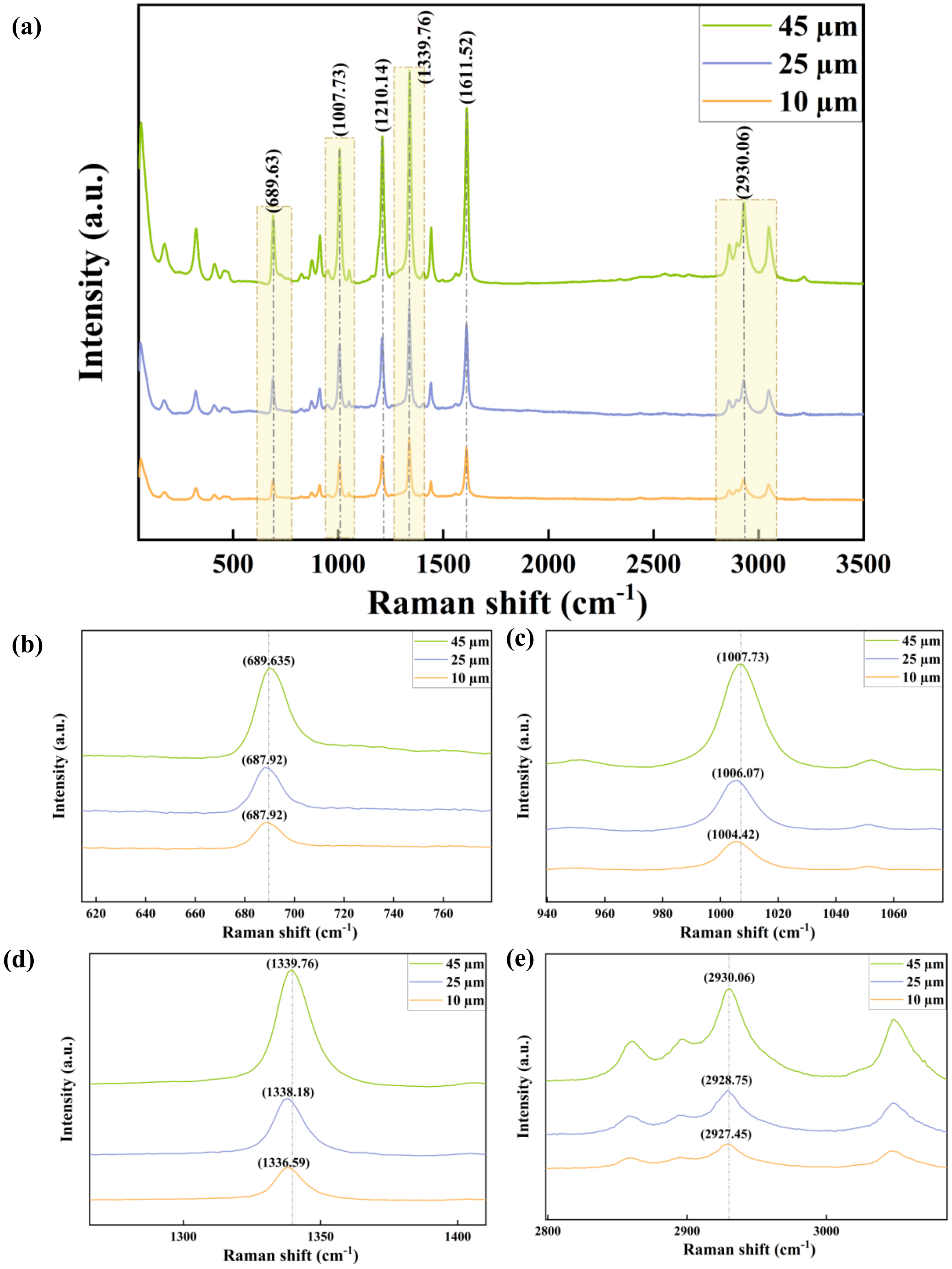

Figure S4. **(a)** Full Raman spectra (range 3500–100 cm^{-1}) of PAC films at thicknesses of 10 μm, 25 μm, and 45 μm. Characteristic peaks are highlighted and marked. Enlarged views of Raman spectra showing detailed shifts and intensities of characteristic Raman peaks at around **(b)** 689.63 cm^{-1} (C–Cl stretching vibrations), **(c)** 1007.73 cm^{-1} (benzene ring symmetric breathing vibrations), **(d)** 1339.76 cm^{-1} (C–H bending vibrations), and **(e)** 2930.06 cm^{-1} (C–H stretching vibrations), respectively.

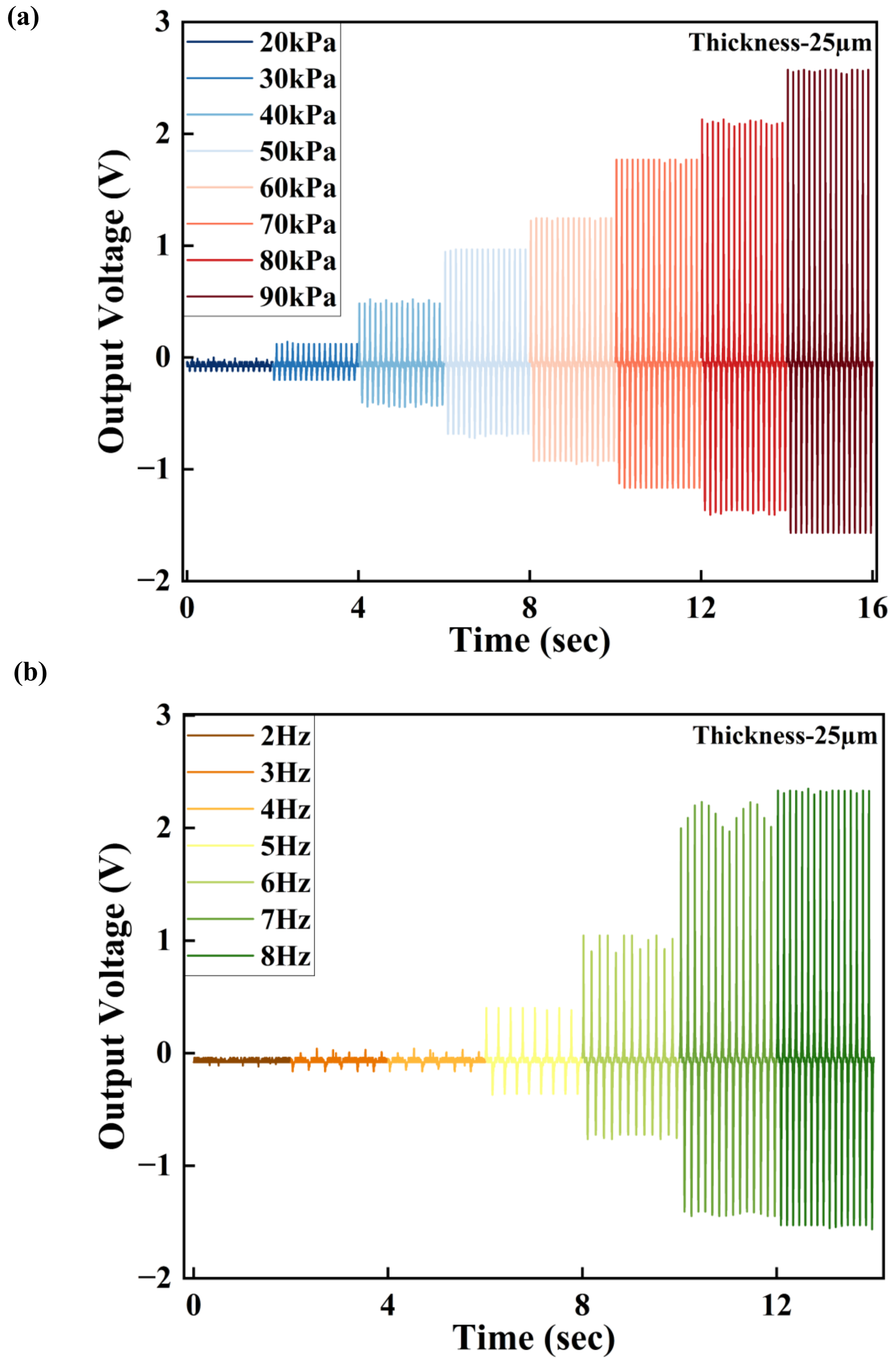

Figure S5. Stepwise incremental responses of the PAC sensor are illustrated in (a) with pressure varying from 20 kPa to 90 kPa at a constant frequency of 8 Hz, and in (b) with frequency varying from 2 Hz to 9 Hz at a constant pressure of 90 kPa.

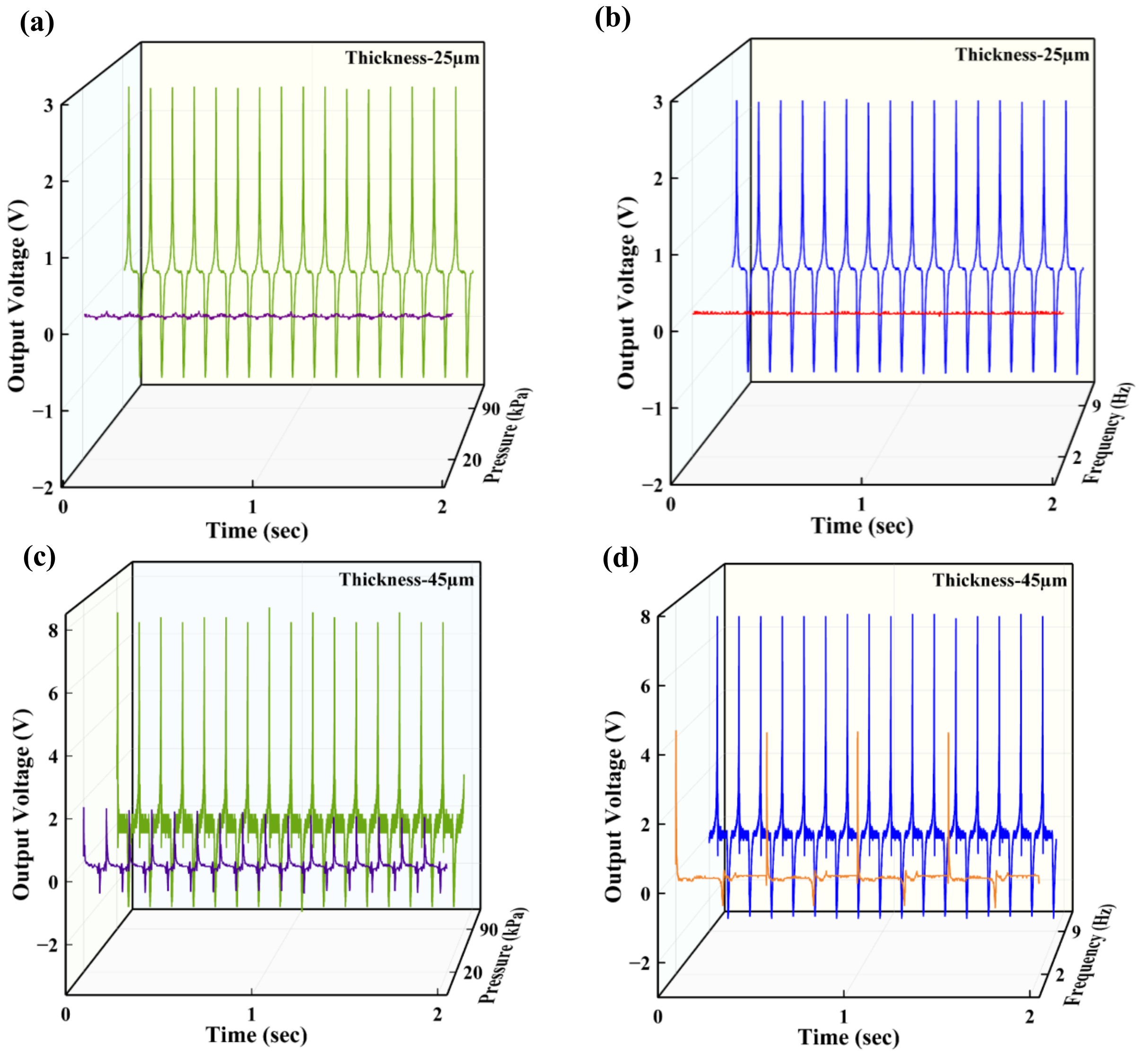

Figure S6. (a) Plot of output voltage versus time for PAC film with 25 μm thickness under applied pressures of 20 kPa and 90 kPa; and **(b)** at frequencies of 2 Hz and 9 Hz; **(c)** corresponding voltage-time characteristics for PAC films with a thickness of 45 μm under identical pressures; and **(d)** under the same excitation frequencies.

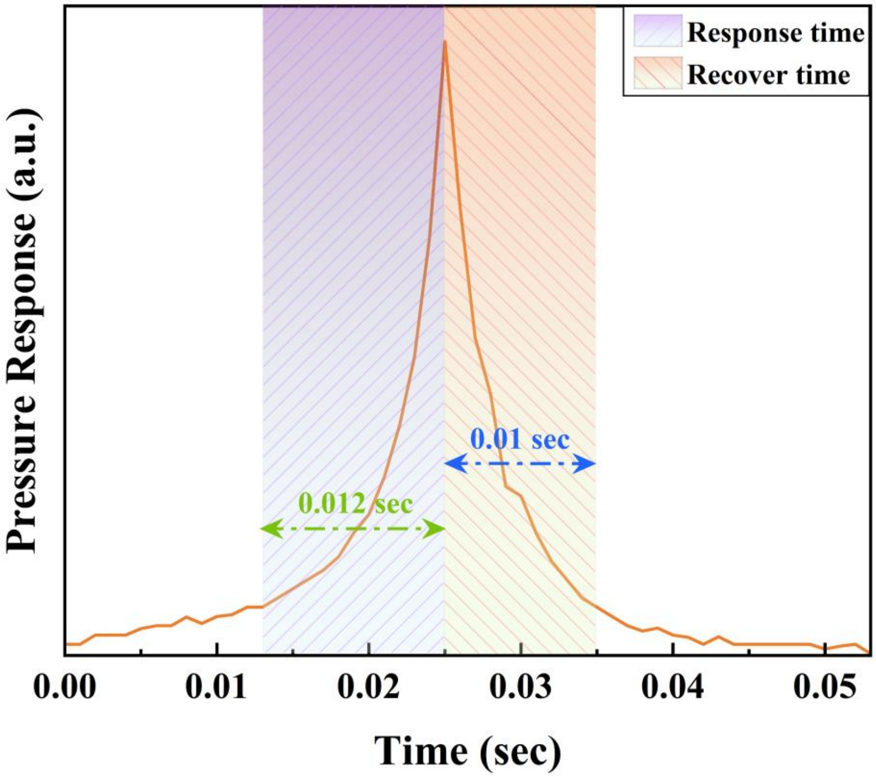

Figure S7. Response and recovery times of the PAC-based piezoelectric pressure sensor under instantaneous pressure loading and unloading conditions.

(a)

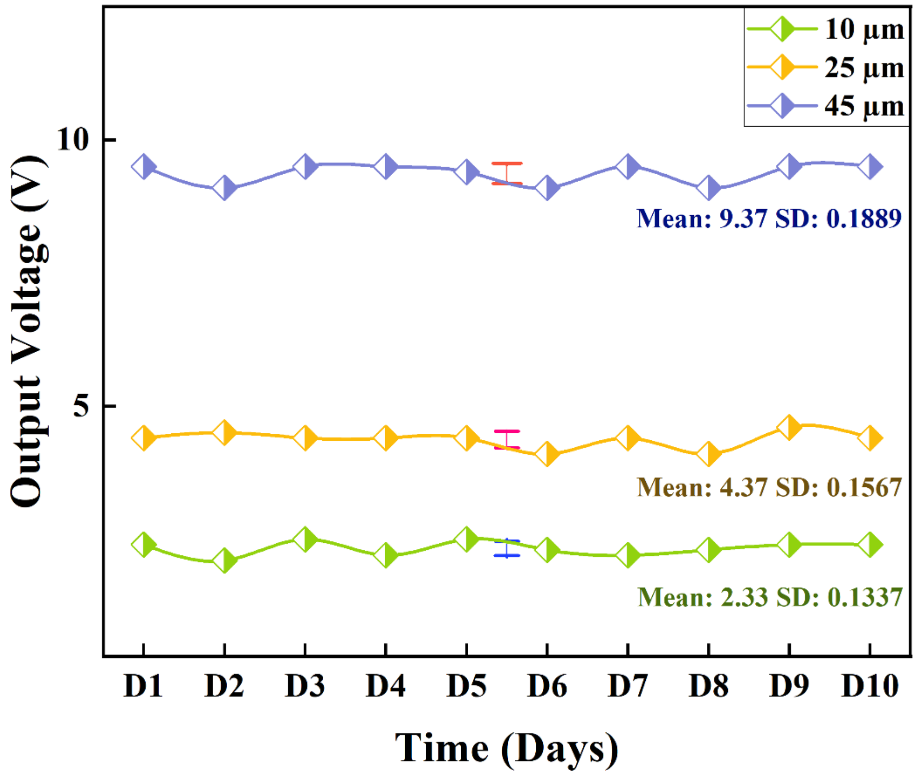
10 µm
25 µm
45 µm
10
5
Output Voltage (V)
Mean: 9.37 SD: 0.1889
Mean: 4.37 SD: 0.1567
Mean: 2.33 SD: 0.1337
D1
D2
D3
D4
D5
D6
D7
D8
D9
D10
Time (Days)

(b)

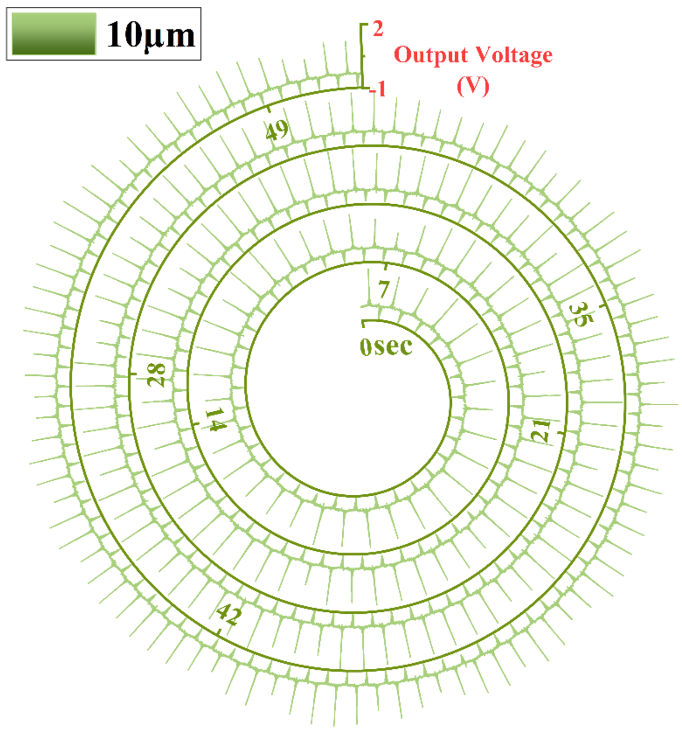
10µm
2
-1
Output Voltage
(V)
0sec
7
14
21
28
35
42
49

(c)

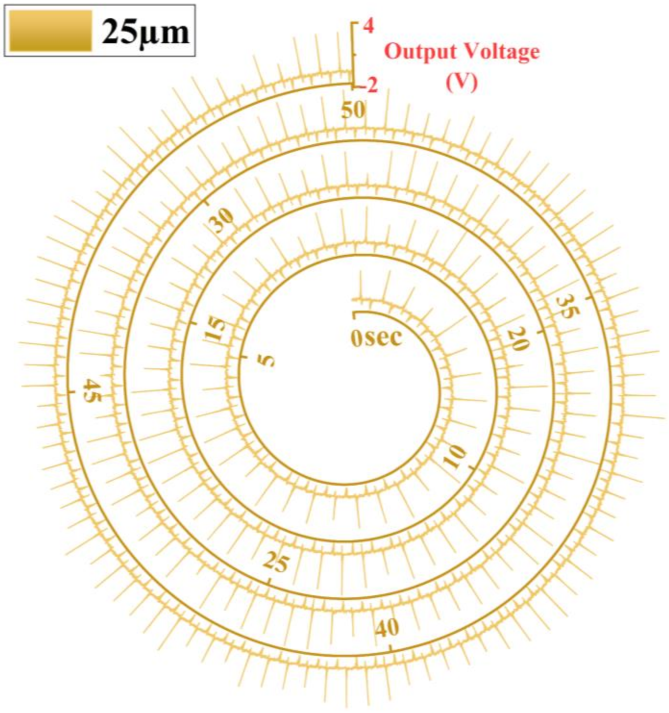

(d)

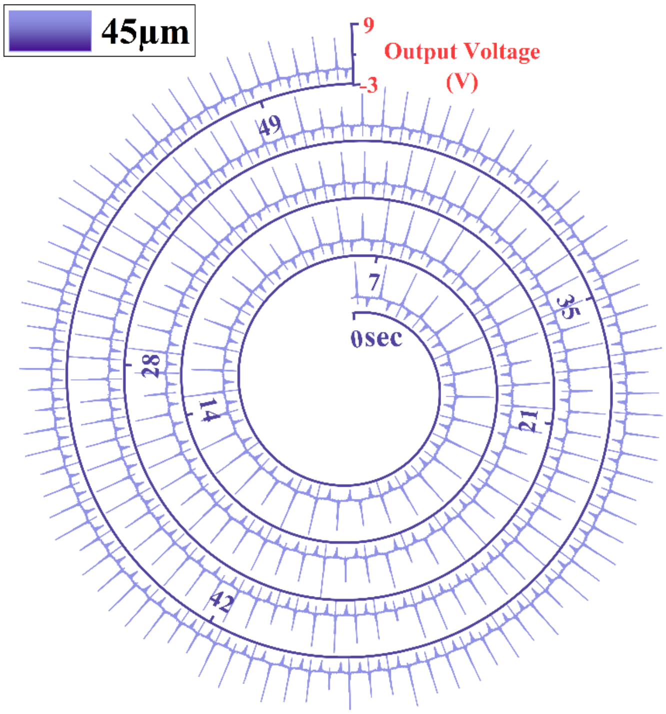

Figure S8. Long-term stability tests of PAC piezoelectric pressure sensors with various thicknesses (10 μm, 25 μm, and 45 μm). **(a)** Daily average output voltage values recorded over a 10-day period under continuous fatigue tests (pressure: 90 kPa, frequency: 5 Hz, temperature: 20–25 °C, humidity: 30–35%). **(b–d)** Corresponding detailed spiral plots illustrating real-time output voltage stability for sensors with thicknesses of **(b)** 10 μm, **(c)** 25 μm, and **(d)** 45 μm, respectively.

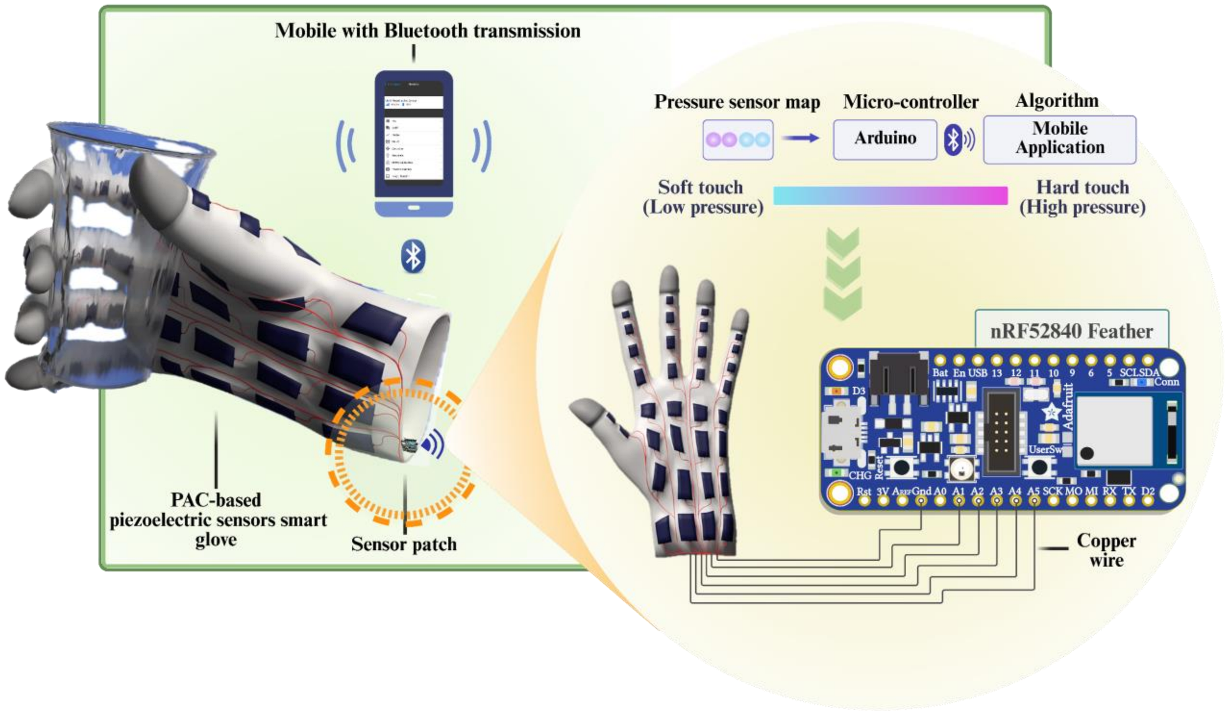

Figure S9. Schematic illustration of the smart glove integrated with a PAC-based piezoelectric pressure sensor array for real-time wireless monitoring. Sensor signals are processed by an Arduino-based nRF52840 SoC via Bluetooth Low Energy (BLE), enabling wireless communication with a mobile application.
